# Tandem duplication within the *DMD* gene in Labrador retrievers with a mild clinical phenotype

**DOI:** 10.1016/j.nmd.2022.08.001

**Published:** 2022-08-06

**Authors:** G. Diane Shelton, Katie M. Minor, Natassia M. Vieira, Louis M. Kunkel, Steven G. Friedenberg, Jonah N. Cullen, Ling T. Guo, Mayana Zatz, James R. Mickelson

**Affiliations:** a Department of Pathology, School of Medicine, University of California San Diego, LaJolla, CA, USA; b Department of Veterinary and Biomedical Sciences, College of Veterinary Medicine, University of Minnesota, Saint Paul, MN, USA; c The Division of Genetics and Genomics, Boston Children’s Hospital, Department of Pediatrics and Genetics, Harvard Medical School, Boston, MA, USA; d Department of Veterinary Clinical Sciences, College of Veterinary Medicine, University of Minnesota, Saint Paul, MN, USA; e Human Genome and Stem Cell Center, Biosciences Institute, University of Sao Paulo, Brazil

**Keywords:** Dog, Myopathy, Muscular dystrophy, Whole genome sequencing

## Abstract

A form of dystrophinopathy with mild or subclinical neuromuscular signs has been previously reported in a family of Labrador retrievers. Markedly and persistently elevated creatine kinase activity was first noted at 6 months of age. Skeletal muscle biopsies revealed a dystrophic phenotype, with dystrophin non-detectable on western blotting and immunohistochemical staining, and with increased utrophin expression. In this report we demonstrate with western blotting that *α*-dystroglycan is present at essentially normal levels. Whole genome sequencing has also now revealed an approximately 400kb tandem genomic DNA duplication including exons 2-7 of the *DMD* gene that was inserted into intron 7 of the wild type gene. Skeletal muscle cDNA from 2 cases contained *DMD* transcripts as expected from an in-frame properly-spliced exon 2-7 tandem insertion. A similar 5’ duplication involving *DMD* exons 2-7 has been reported in a human family with dilated cardiomyopathy but without skeletal myopathy. This is the 3^rd^ confirmed mutation in the *DMD* gene in Labrador retrievers.

## 1. Introduction

Variable severity of clinical phenotypes in canine models of human dystrophin deficient muscular dystrophy is well known and may confound analysis of results in pre-clinical therapeutic trials [1]. In addition to severe phenotypes in dystrophin deficient Labrador retrievers [2], mild clinical presentations have also been identified. A Becker-like phenotype was described in 2014 in a 3-year-old Labrador retriever with mild clinical signs [3] and in 2015 a family of Labrador retrievers with three generations of affected male dogs was reported with undetectable to mild clinical weakness [4]. A neuromuscular disease was suspected in this latter family of dogs based on persistently and markedly elevated creatine kinase (CK) activities when first tested at 6 months of age. Histopathology of muscle biopsies was consistent with a dystrophic phenotype, and dystrophin protein was absent on western blot analysis. Immunofluorescence staining of muscle cryosections detected dystrophin protein only in revertant fibers [4]. However, PCR-based Sanger exon sequencing did not detect any associated dystrophin (*DMD*) gene variants in this family of dogs.

There are currently two variants in the *DMD* gene reported to cause dystrophin deficient MD in Labrador retrievers: these include a rearrangement involving the 3’ end of intron 20 [2] and a pseudoexon insertion in intron 19 leading to a premature stop codon [5,6]. In addition, polymorphisms in suspected modifier genes including *Jagged1* in mildly affected golden retriever dogs [7] and *LTBP4* in human DMD patients [8], are thought to influence the clinical dystrophic phenotype. *PITPNA* may also lead to “escaper” phenotypes in golden retriever dogs and zebrafish [9].

The purpose of this study was to determine whether the molecular basis of the mild form of MD seen previously in a line of Labrador retrievers [4] lies in a yet undetected variant in the *DMD* gene, or whether potential variants in modifier genes would explain the milder course in these dogs.

## 2. Methods

### 2.1. Animals

The mildly affected Labrador retrievers and unaffected relatives characterized in a previous publication [4] were included in this study. DNA was isolated from either whole blood or frozen archived muscle obtained from diagnostic muscle biopsy samples. Muscle or venous blood samples for DNA isolation from 13 affected male dogs in the original study pedigree, 1 unaffected related male dog, 4 normal related female dogs, and 5 other apparently unrelated dogs, were obtained under University of Minnesota IACUC protocol 1903-36865A. Breeders, owners, and veterinarians that treated affected dogs in the original publication were contacted for available clinical outcome that included progression of weakness, information on lifespan and/or euthanasia from unrelated causes.

### 2.2. Whole genome sequencing and analysis

**Fig.1.**
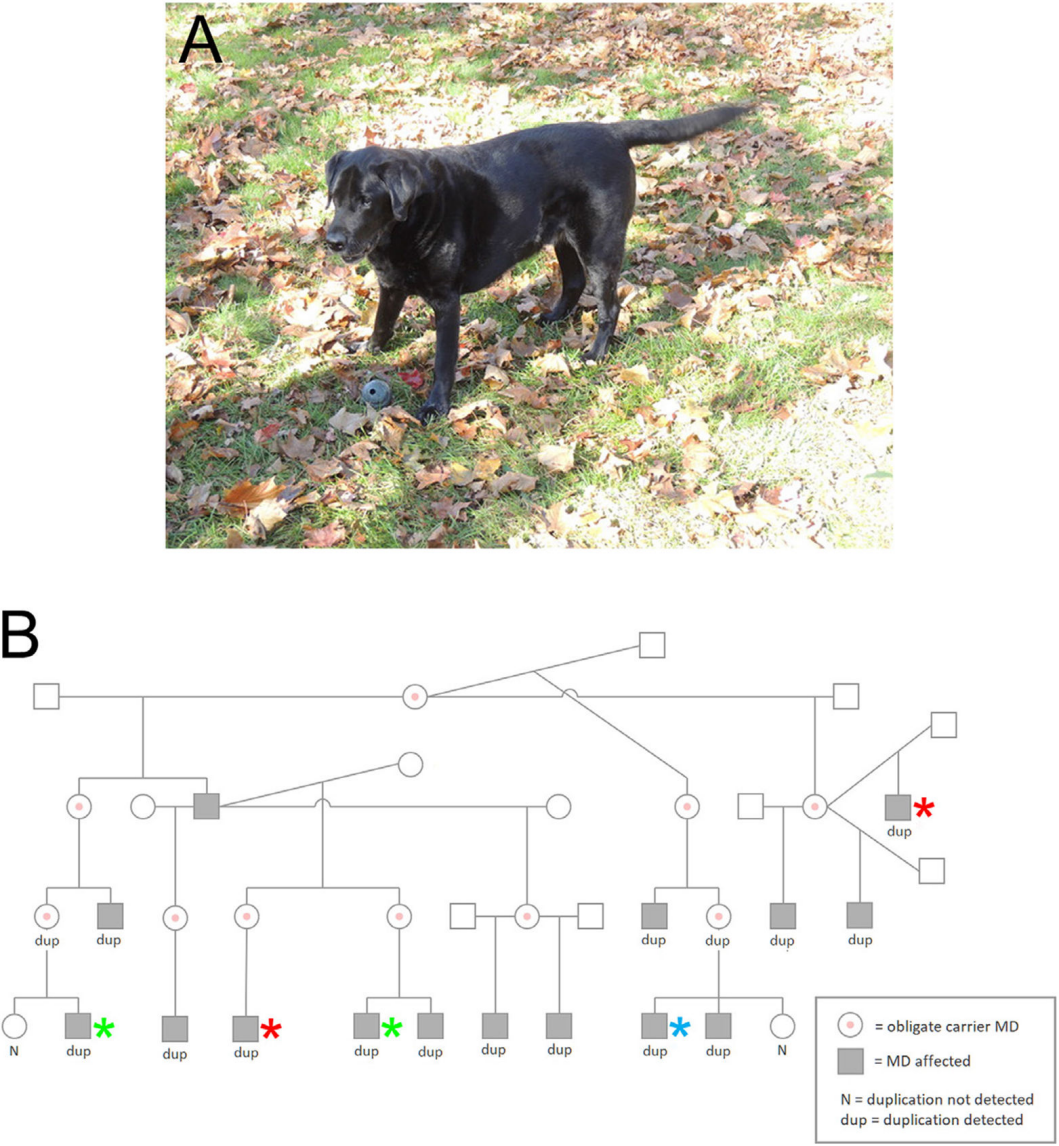
Mildly Affected Labrador Retriever and Pedigree With Genotypes. A. An 11-year-old male Labrador retriever (highlighted by blue asterisk in B) with mild XLMD. B. Pedigree of Labrador retriever family with XLMD and a mild phenotype. Cases used for WGS are highlighted with red asterisks. Cases used for western blot are highlighted with green asterisks. Affected male dogs are identified by the filled square boxes and unaffected males are identified with clear boxes. Carrier female dogs are indicated by circles with central red dots.

**Illumina 125 base pair, paired-end whole genome sequences (WGS) from 2 mildly affected dystrophic Labradors (**Fig. 1, dogs sequenced highlighted with red asterisks) and 1 clinically normal control (from same breeders but not closely related) were generated at the Broad Institute (Boston MA). Sequence reads for all three dogs required repair with the BBMap repair.sh method prior to processing (https://github.com/BioInfoTools/BBMap). The reads were subsequently mapped against the dog reference genome assembly (CanFam3.1) as described [10,11] and are available in NCBI’s Short Read Archive (https://www.ncbi.nlm.nih.gov/sra/PRJNA796268). The WGS case data were compared to those of control genomes from the University of Minnesota’s private WGS database containing 523 dogs of 55 diverse breeds (including 16 Labrador retrievers from unrelated projects) in a search for simple coding variants in the *DMD* gene, as well as the reported modifier genes *Jagged 1, LTBP4* and *PITPNA*. Ultimately, visual analysis of sequence read alignments at the *DMD* locus (chrX:26,288,910-28,335,720) was utilized to search for regions of unpaired reads suggestive of large structural variants.

### 2.3. DMD cDNA and transcript analysis

RNA was isolated from frozen muscle via Trizol reagent (ThermoFisher Scientific) and cDNA prepared from 2 affected and 1 control dog using iScript cDNA Synthesis Kit (Bio-Rad) according to manufactures’ instructions. PCR primers (Table S1) were designed to search for cDNAs containing transcript isoforms that could result from the normal and newly discovered variant *DMD* genes.

### 2.4. DMD variant genotyping

PCR primers flanking the 5’ and 3’ genomic DNA insertion sites that enabled genotyping of the control and variant DMD genes are also supplied in Table S1.

### 2.5. Western blot

To determine the presence or absence of *α*-dystroglycan [*α*-DG] in skeletal muscle from two mildly affected Labradors (green asterisks in Fig. 1B) and an archived age and breed matched control muscle, 20 mg of skeletal muscle were homogenized in 200 μl cell lysis buffer (Cell signaling Cat# 9803) with PMSF and protease inhibitor cocktail (Cell Signaling Cat #5871). Following centrifugation, soluble proteins were resolved using NuPage 4-12% Bis-Tris gels (Invitrogen Cat #NP0321), then transferred to nitrocellulose membranes (Thermo Scientific Cat# 88018). Membranes were blocked for 2 hours at RT with 5% powdered non-fat milk in T-BST and then incubated overnight at 4°C with the following blocking buffer diluted antibodies: human *α* and *β* dystroglycan (1:1000, R&D Systems Cat # AF6868) and *α*-tubulin (1:1000, Sigma Cat # T6074). After washing in T-BST buffer, the membrane was incubated overnight at 4°C with blocking buffer diluted secondary antibodies: anti-sheep HRP antibody (1:5000, R&D Systems Cat # HAF016) and anti-mouse HRP antibody (1:5000, Jackson ImmunoResearch Cat # 115-035-62). After washing in T-BST buffer, the protein bands were visualized using SuperSignal West Dura kit (Thermo Scientific, Cat #37071).

## 3. Results

### 3.1. Animals and long-term outcome

The mild clinical presentation, histopathological findings, and absence of dystrophin protein in muscle by immunofluorescent staining and western blotting have been previously described [4]. An error was noted in the original paper regarding Dog 14 (Fig. 1B,pedigree) as the dog was in fact affected and not wild-type. Fig. 1B in this current manuscript represents the correct pedigree. Long-term outcome has now been determined on 13 related clinically affected male Labrador retrievers. Four dogs died acutely of cardiomyopathy and arrhythmias at 4-5 years of age, two dogs were euthanized at 6 and 7 years of age for disseminated neoplasia (aggressive metastatic sarcoma, mast cell tumor), and the remaining 7 dogs were either euthanized for age related causes or were still alive at 11-12 years of age (Fig. 1A).

### 3.2. Detection of a Structural Variant

**Fig. 2.**
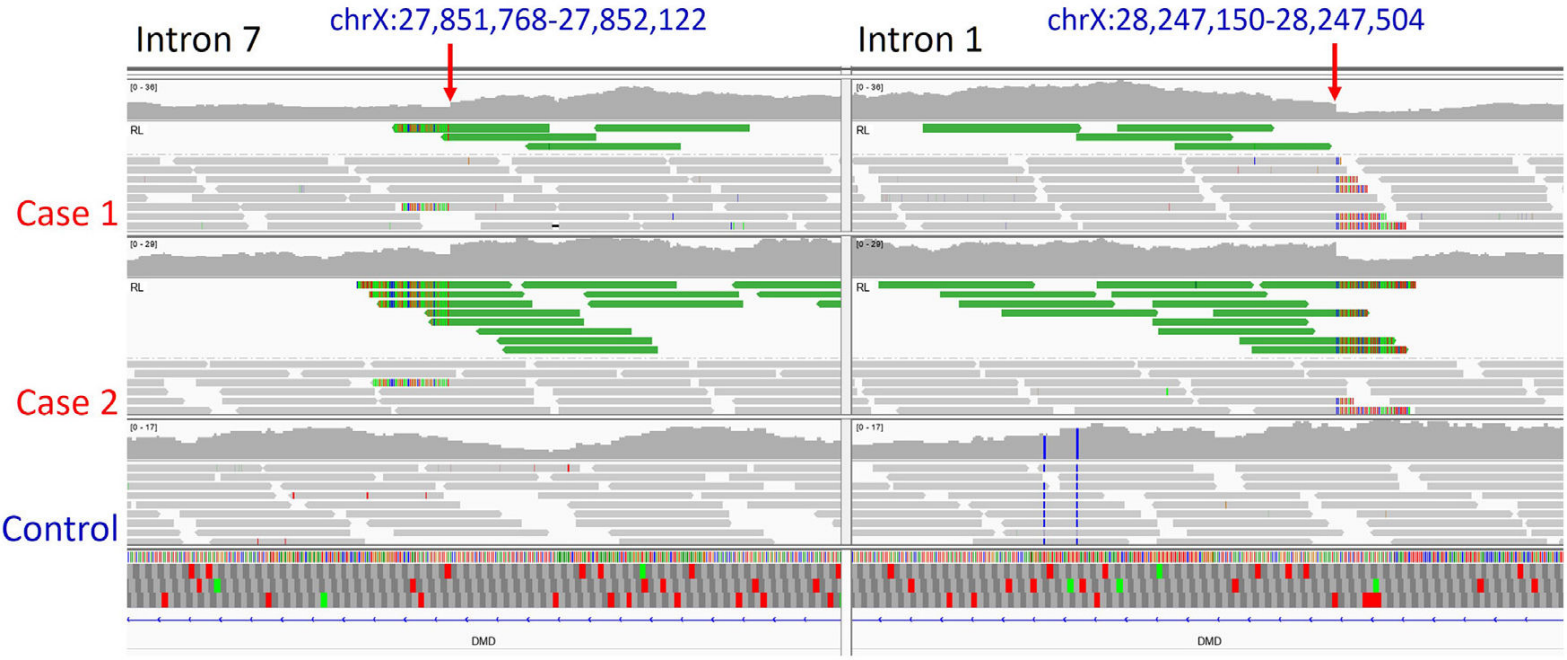
Whole Genome Sequence Reads From 1 Control and 2 Cases Mapped to the *DMD* Gene. Indicated bp positions within intron 7 (left) and intron 1 (right) are shown. In each of the cases there are WGS sequence reads (green bars flanked by improperly aligned bases at the breakpoint)) in which the corresponding mate pair does not align as expected. Grey bars are mate pairs that align appropriately. Red arrows indicate the sites for which flanking PCR primers were designed to evaluate the insertion sites. The genomic regions containing the green reads also demonstrate an increase in the depth of read coverage. WGS alignments from a control Labrador retriever do not display this pattern.

**Fig. 3.**
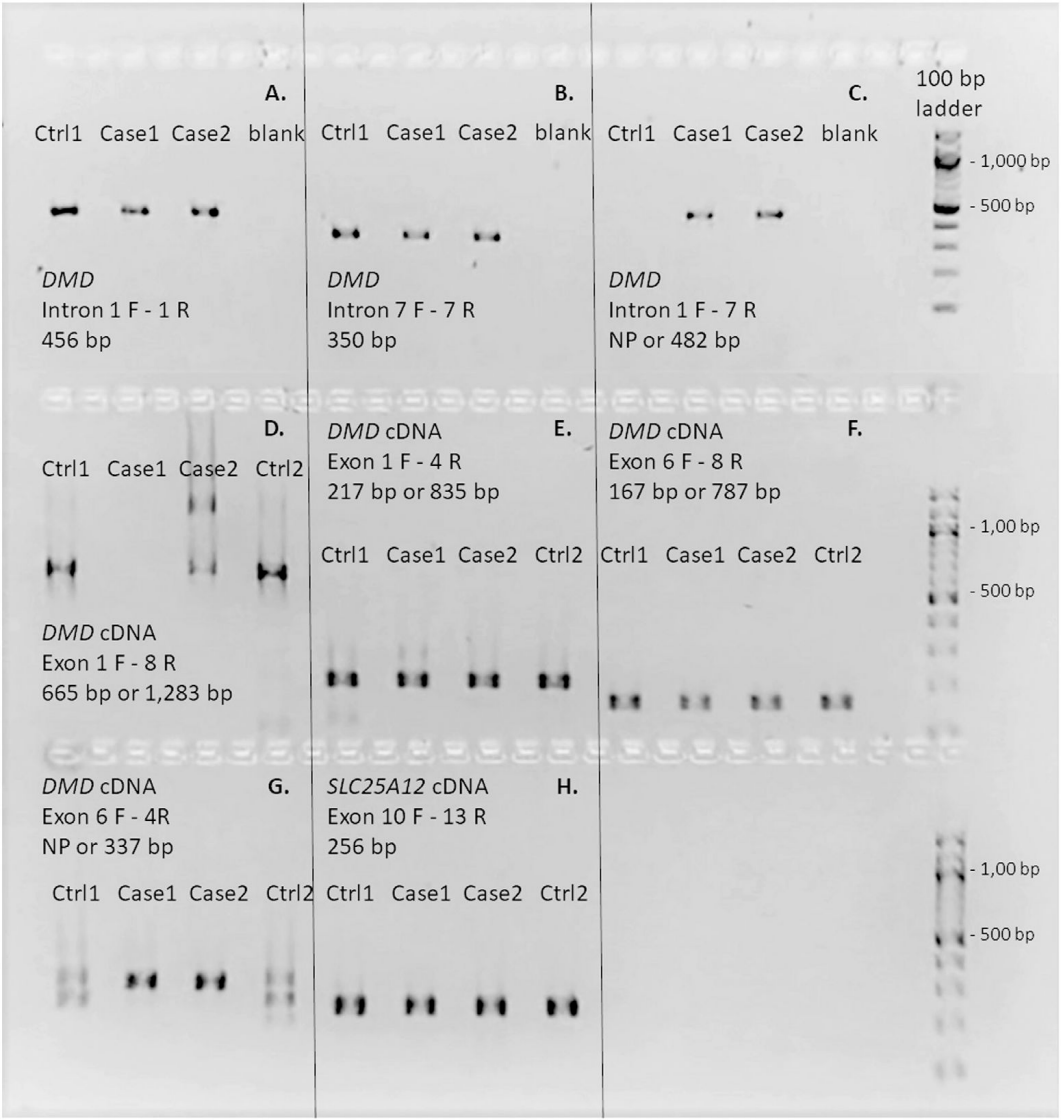
Genomic structure of the *DMD* gene variant and the mRNA derived from it. Panels A - C. Three primer combinations, based within introns and indicated in each panel (sequences provided in Table S1) were designed to test for the presence of specific genomic DNA segments in the case and control DNAs. Panels D – G. Four primer combinations, bases within exons and indicated in each panel (sequences provided in Table S1) were designed to test for the presence of specific segments in case and control cDNAs. Panel H. A control reaction with primers for the *SLC25A12* gene.

Fig. 2 presents WGS read alignments from the affected and control dogs at two locations along the *DMD* gene locus. Significant numbers of unpaired reads at the chrX:27,851,768-27,852,122 bp position (Intron 7, left hand panel) and chrX:28,247,150-28,247,504 bp position (intron 1, right hand panel) indicated the possible presence of an insertion. PCR-based Sanger sequence analyses were performed utilizing primers designed to flank both potential insertion points (red arrows). Primer pairs flanking the putative intron 1 insertion point produced an amplicon of the expected size from both case and control DNAs, as did primer pairs flanking the putative intron 7 insertion site (Fig. 3A and B, respectively). However, a primer pair consisting of an intron 1 forward primer and an intron 7 reverse primer flanking the putative insertion point produced an amplicon with *DMD* intron 1 and intron 7 sequences in the cases, but no product in the controls (Fig. 3C). This result is consistent with a tandem duplication of the *DMD* gene segment comprising introns 1 through 7 of approximately 400 kb in length. The identical duplication and insertion were confirmed by genotyping 11 additional related cases (shown in Fig. 1). Two female carriers were also identified. No SNPs or small indels in possible modifying genes that included *LTBP4, Jagged1* and *Pitpna* were found in the WGS.

### 3.3. Transcript analysis

**Fig. 4.**
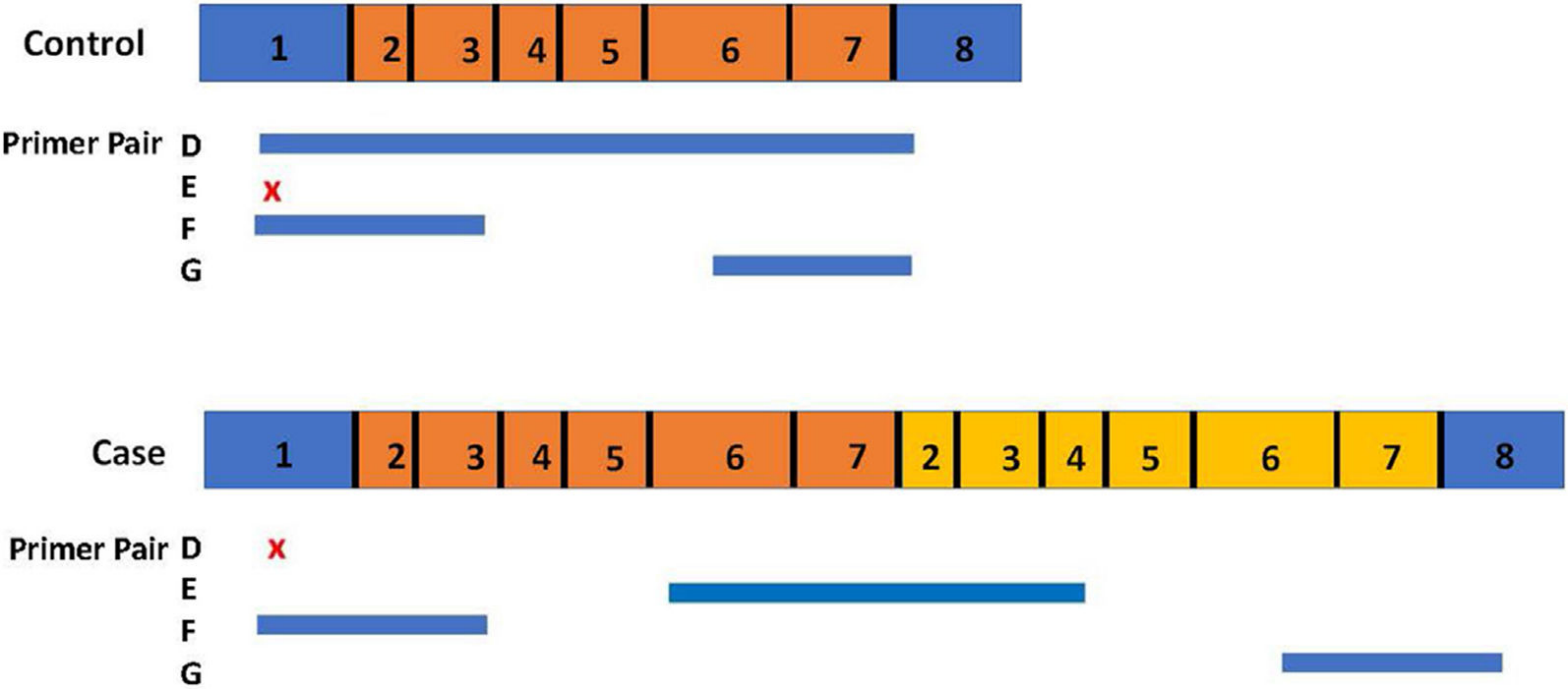
Model of the *DMD* mRNA. Results of the transcript analysis of Fig. 3 indicate that a tandem duplication and insertion within the *DMD* gene results in an mRNA containing in frame exons 2-7 (yellow) placed directly 3’ of the normal exon 1 – 7 segment (orange) and directly preceding exon 8 (blue). Four primer combinations (D – G; sequences provided in Table S1) were designed to test for the presence of specific cDNA segments in the case and control cDNAs. A blue bar indicates a successful PCR reaction from that primer pair and a red x indicates no PCR product was produced from that primer pair.

PCR primers based on the predicted cDNAs were also designed to better define the 5’ exonic structures of the case and control *DMD* gene transcripts (Fig. 3D-G). As expected, amplicons containing contiguous sequence of *DMD* exons 1-8 (3D), exons 1-4 (3E), and exons 6-8 (3F) were generated from the control cDNA. Similar analyses with the case cDNA also detected the expected exons1-4 (3E) and exons 6-8 (3F) amplicons. However, the *DMD* exon 1-8 primers generated no, or variable amplicon sizes in the case cDNAs, including a normal-sized exon 1-8 product (3D), a longer exon 1-8 product containing tandem inserted exonic sequence (3D), and sometimes low inconsistent levels of other intermediate-sized products that varied by experiment (not shown). Further, an amplicon derived from *DMD* exon 6 to exon 4 was identified in the cases but not the control (3G). All but the faint intermediate-sized products from the case cDNA were confirmed by Sanger sequencing. These are the results expected from transcription and proper splicing of inserted *DMD* genomic DNA containing exons 2-7. Sanger sequencing of these amplicons also revealed the inserted sequence to be in-frame and therefore not by itself disruptive to translation into a protein. A proposed model of the structure of the mRNA resulting from the tandem duplication and insertion is provided in Fig. 4.

### 3.4. Western blot

In the previous human case report describing the 5’ dystrophin duplication mutation [12], antibodies against *α*-DG protein detected a mild to moderately reduced abundance of protein in both skeletal muscle and heart. For comparison, a western blot was performed on remaining skeletal muscle biopsy material from 2 mildly affected Labradors and an archived control muscle (Supplemental Fig. 1). Compared to control muscle, the protein abundance of *α*-DG from the affected dogs did not appear reduced.

## 4. Discussion

This study expands the spectrum of dystrophin gene mutations in Labrador retriever X-linked muscular dystrophy and identifies a variant associated with mild clinical signs. Of the 13 mildly affected and genotypically confirmed male dogs, 7 dogs lived a relatively normal lifespan with a good quality of life as reported by the owners. Of interest, 4 affected dogs died acutely of cardiomyopathy and arrhythmias. A similar 5’ *DMD* duplication was found in a human DMD patient with cardiomyopathy but without clinically described muscle weakness [12]. In the human case as in the mildly affected dogs, a lack of detectable dystrophin protein was found on western blot, and except for revertant fibers, sarcolemmal membrane protein localization was absent by immunohistochemistry. A complete cardiac evaluation would be indicated in the future for dogs affected with this variant to fully characterize involvement of the heart in the clinical phenotype.

In our study, a structural variant was identified in affected Labrador retriever dogs (Figs. 2 and 3) consisting of a tandem insertion of an approximately 400 kb *DMD* gene fragment containing exons 2-7 and the inclusive introns. Exons 2-7 encode the N-terminal actin binding domain of dystrophin. This duplicated and inserted *DMD* allele can apparently be transcribed into an mRNA with the indicated repeat of exons 2-7 detectable by RT-PCR. Our detection of several cDNAs apparently generated from rare splicing events within the duplicated segment is consistent with the findings from the human cardiomyopathy study where the causative variant also was a tandem duplication involving exons 2-7 [12]. Missense mutations in ABD1 may cause loss of dystrophin function via protein instability and aggregation rather than through loss of ligand binding function [13,14]. More severe disease progressions may be due to the combinatorial effects of some mutations on both protein aggregation and impaired actin-binding activity. We are uncertain if reference to missense mutations can be applied to our case of a tandem repeat mutation that, if translated into protein, would be in frame. Whether such a protein would be stable or degraded is an open question.

Tandem duplication mutations such as this may be the direct cause of many rare inherited diseases and have been reported to account for up to 10% of cases [15]. Animal models with such tandem duplication mutations are limited [16]. This report is the first instance of a tandem duplication in the dystrophin gene resulting in a dystrophinopathy in dogs.

Intragenic duplications have been implicated in DMD in people [17]. In one large study of 7,149 *DMD* mutations using the TREAT-NMD DMD Global database (http://umd.be/TREAT_DMD/), duplications accounted for 11% of the total mutations with duplication of exon 2 the most frequent [17]. Typically, intragenic duplications cause a disruption of the open reading frame and loss of function of the affected protein. However, in this family of dogs, with this specific *DMD* variant, the open reading frame was not disrupted. Duplications expected to shift the translational reading frame generally result in a more severe clinical phenotype than duplications that maintain the reading frame [18]. In the TREAT-NMD DMD global database, 7% of the total *DMD* duplication mutations did not follow the reading-frame rule [17]. Nevertheless, dystrophin protein was not detected in the affected dog muscles [4]. We speculate that, although detectable by RT-PCR, there may not be a large concentration of the spliced inserted mutant *DMD* mRNA present in vivo due to degradation, a small amount of functional DMD protein could be made from the limited amount of appropriate mRNA, but not be detectable on western blots or immunohistochemistry, or that any DMD protein produced with the in-frame insertion could be misfolded and degraded. Low- level dystrophin expression has been shown to improve mechanical properties and function of dystrophic muscle in the mdx-Xist [19] and mdx-4cv [20] mouse models.

Mild to moderately reduced amounts of *α*-DG protein were found in heart and skeletal muscle using whole tissue homogenates in western blots in a human case with a 5’ dystrophin duplication mutation [12]. We did not find reduced *α*-DG in a western blot from 2 mildly affected Labradors. In the human case, however, microsomal membrane preparations from heart muscle suggested that although *α*-DG is present in the cell, cardiac dystrophin deficiency results in a poor association of a-DG with other membrane protein complexes. Sufficient biopsy material from skeletal muscle did not remain for further analysis in the affected dogs and no heart tissue was available.

Previous studies using Sanger sequencing of the *DMD* gene cDNA from dogs in this pedigree did not identify potentially functional variants. We consider it likely that this approach designed to detect simple variants, was not sensitive enough to detect a structural variant that was inserted within introns and distantly removed from the intron-exon boundaries. As a result, we applied WGS that could detect a structural variant encompassing non-coding sequence and confirmed it with a PCR analysis of both ends of the duplicated insertion. We conclude that WGS analysis coupled with appropriate PCR confirmation of the duplication boundaries is often necessary to comprehensively search for causative non-coding large structural variants.

Heterogeneity in clinical severity varying from severe disease to occasionally mild disease has been described in colonies of golden retrievers with X-linked MD resulting from a splice site point mutation and skipping of exon 7 [1, 21], and in a colony of Labrador retrievers with X-linked MD and a 22 Mb inversion within exon 20 [2] leading to difficulties in interpretation of results of pre-clinical trials. For this reason, polymorphisms in modifier genes that could ameliorate severe clinical phenotypes have been investigated. Modifier genes including *LTBP4* in DMD patients [8], and *Jagged1* [7] and *Pitpna* [9] in canine XLMD have been reported to result in mild clinical “escaper” phenotypes. Upregulation of *Lama1* in muscle was shown to prevent muscle fibrosis and weakness in the murine model dy^2j^/dy^2j^ of laminin *α*2 deficient congenital muscular dystrophy [22]. In the Labrador retrievers of this report, the clinical phenotype was mild among all the affected dogs without any severely affected dogs, making an effect of a modifier gene unlikely. Polymorphisms in these possible modifying genes were not found in this family of Labrador retrievers by analysis of SNPs and small indels in the WGS. We conclude that the tandem duplication in the *DMD* gene is the likely cause of the mild myopathic phenotype in this family of Labrador retrievers. Furthermore, the mild phenotype in these dogs represents an additional example of a functional muscle in a large animal despite dystrophin deficiency opening new perspectives for DMD therapeutic trials

## 5. Conclusions

This study expands our knowledge of known *DMD* variants affecting the Labrador retriever breed and with direct correlations to the corresponding human conditions. This is also an important example of a large tandem duplication in *DMD* in the dog that is the likely cause of a mild clinical phenotype.

## Supplementary Material

1 Supplementary Figure 1. Western blot analysis of skeletal muscle extracts from 2 cases of Labrador retrievers with mild dystrophin deficient muscular dystrophy and an archived muscle from a close to age and breed matched control dog. Blots were incubated with antibodies against *α* and *β*-dystroglycan, and *α*-tubulin as a loading control.

2
