# Supplementary figures and images for: Tandem duplication within the DMD gene in Labrador retrievers with a mild clinical phenotype
Source: Neuromuscul Disord. Author manuscript; Available in PMC 2023 Mar 26. (PMC10040250; doi:10.1016/j.nmd.2022.08.001)

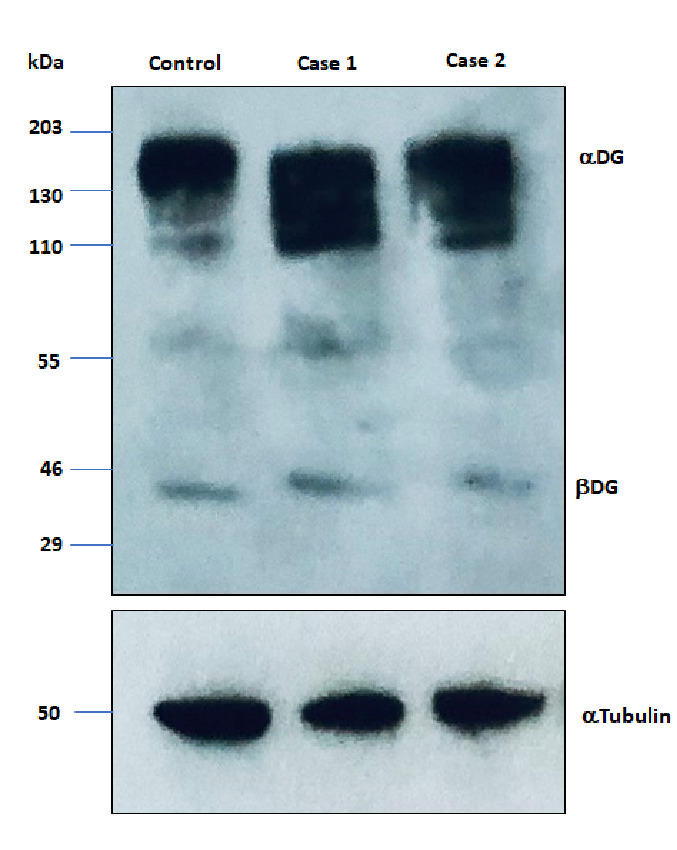

Supplement: 1 — Supplementary Figure 1. Western blot analysis of skeletal muscle extracts from 2 cases of Labrador retrievers with mild dystrophin deficient muscular dystrophy and an archived muscle from a close to age and breed matched control dog. Blots were incubated with antibodies against α and β-dystroglycan, and α-tubulin as a loading control. [file NIHMS1883753-supplement-1.jpg]
